# Emergency Services Capacity of a Rural Community in Guatemala

**DOI:** 10.5811/westjem.2022.7.56258

**Published:** 2022-09-12

**Authors:** Matthew Hughes, Jessica Schmidt, James Svenson

**Affiliations:** University of Wisconsin School of Medicine and Public Health Department of Emergency Medicine, Madison, Wisconsin

## Abstract

**Introduction:**

Access to emergency care is an essential part of the health system. Improving access to emergency services in low- and middle-income countries (LMIC) decreases mortality and reduces global disparities; however, few studies have assessed emergency services resources in LMICs. To guide future improvements in care, we performed a comprehensive assessment of the emergency services capacity of a rural community in Guatemala serving a mostly indigenous population.

**Methods:**

We performed an exhaustively sampled cross-sectional survey of all healthcare facilities providing urgent and emergent care in the four largest cities surrounding Lake Atitlán using the Emergency Services Resource Assessment Tool (ESRAT).

**Results:**

Of 17 identified facilities, 16 agreed to participate and were surveyed: nine private hospitals; four public clinics; and three public hospitals, including the region’s public departmental hospital. All facilities provided emergency services 24/7, and a dedicated emergency unit was available at 67% of hospitals and 75% of clinics. A dedicated physician was present in the emergency unit during the day at 67% of hospitals and 75% of clinics. Hospitals had a significantly higher percentage of available equipment compared to clinics (85% vs 54%, mean difference 31%; 95% confidence interval (CI) 23–37%; P = 0.004). There was no difference in availability of laboratory tests between public and private hospitals or between cities. Private hospitals had access to a significantly higher percentage of medications compared to clinics (56% vs 27%, mean difference 29%; 95% CI 9–49%; P = 0.024).

**Conclusion:**

We found a high availability of emergency services and universal availability of personal protective equipment but a severe shortage of critical medications in clinics, and widespread shortage of pediatric equipment.

## INTRODUCTION

Emergency services are an essential part of the health system and serve as the first point of contact for many around the world. It is estimated that emergency services as defined by the World Health Organization (WHO)^1^ could directly impact over half of the mortality in low- and middle-income countries (LMIC)^2^; improving emergency services has been shown to lead to decreases in mortality.^2^,^3^ For over a decade, there has been a growing international focus on improved access to trauma and emergency services starting with the World Health Assembly Resolution of 2007 (WHA60.22).^4^ However, disparities in access to and availability of emergency services still exist and are accentuated in LMICs.^5^

### Importance

Our study focuses on rural Guatemala, which provides its own unique healthcare challenges. Overall, half of Guatemala’s population is indigenous.^6^ Despite having the biggest economy in central America, Guatemala has one of the highest inequality rates in Latin America and ranks among the worst countries in the Central American region for several major health indicators.^7^,^8^ Emergency services in Guatemala are still in the early stages. There are few organized prehospital services and most people have to rely on public or self-transport to access emergency care.^9^ Although previous work has evaluated hospitals in urban areas,^10^ and focused studies on limited scopes of service such as trauma or surgery have been done,^11^–^15^ the ability to provide a standardized set of emergency services, or “emergency services capacity” in rural areas of Central and South America has not been systematically studied.

### Goals of This Investigation

In this study our purpose was to assess the emergency services capacity of a rural community in Guatemala serving a mostly indigenous population, using a tool adapted to acute care settings in LMICs.

## METHODS

### Study Design and Setting

This is a cross-sectional study of healthcare facilities in San Lucas Tolimán and the other three largest cities surrounding Lake Atitlán in the Sololá department of Guatemala. San Lucas Tolimán is situated in South-Central Guatemala on the shores of Lake Atitlán. The town and its surrounding communities are home to a mostly indigenous, highland Mayan population. A recent history of civil war and genocide has left these mountain villages impoverished, with entrenched cultural and socioeconomic barriers limiting access to education, basic sanitation, and healthcare.^16^,^17^ San Lucas Tolimán has a population of 17,000 people living in the town proper, with an additional 14,000 people spread among 19 surrounding rural communities. The average yearly income is less than 1,000 US dollars (USD), or the equivalent of $3 USD per day. An established health promoter program, managed by the central San Lucas Tolimán hospital, helps to provide basic medical care and health education to neighboring communities.^18^

Guatemala has a nationalized healthcare system that is free to all citizens (Ministry of Public Health and Welfare). There is also a system of clinics and hospitals available to government and non-government salaried employees and their families (Guatemalan Institute of Social Security, or IGSS in Spanish).^19^

### Selection of Participants

We used the snowball method to establish an exhaustive sample of public and private healthcare facilities that provide urgent or emergent care in the cities of Sololá, Panajachel, Santiago Atitlán, and San Lucas Tolimán. Facilities included public hospitals, private hospitals, and public clinics. Facilities providing care within the nationalized system of IGSS were considered public institutions for this study. Other facilities, including private for-profit, non-governmental, traditional medicine practitioners, and missions were considered private institutions.^19^ A hospital was defined as a facility designed to care for at least one patient overnight. We included clinics in this study due to the local practice of patients presenting first to their nearest clinic for even life-threatening conditions and from there being transferred by ambulance or private vehicle to a higher level of care.

**Population Health Research Capsule**

What do we already know about this issue?

*Prior work has focused on the emergency services capacity of countries on the African continent, but only one small study has been performed in Latin America*.

What was the research question?

*What is the emergency services capacity of a rural region in Guatemala?*

What was the major finding of the study?

*We found a widespread lack of pediatric equipment and large gaps in basic supplies in clinics*.

How does this improve population health?

*This is the most comprehensive study of emergency services capacity in Latin America to date and offers suggestions for capacity improvement in similar communities*.

### Data Collection

We identified the medical director of each facility who was informed of the study protocol and given a copy of the survey tool. Verbal consent was obtained from each facility’s medical director. A single, bilingual investigator performed all surveys in Spanish through in-person site visits lasting one to three hours, which consisted of interviews of facility staff, direct visual inspection of medications and equipment, and review of documents regarding staffing and available services. Facility staff interviewed consisted of at least the medical director and the emergency unit charge nurse, as well as on occasion financial administrators and various technicians when primary interviewees were unable to answer a question. We conducted the survey in January 2020 employing the Emergency Services Resource Assessment Tool (ESRAT), developed by the Strengthening Emergency Systems Program team of the Columbia University Mailman School of Public Health. The tool is well adapted to our study setting as it has been previously used in a Central American setting and is available in Spanish.^10^

The ESRAT uses key informant interviews and direct inspection of logs, medications, and equipment to assess a healthcare facility’s ability to address 76 quality indicators related to seven clinical conditions (trauma, sepsis, acute respiratory compromise, shock, altered mental status, pain, and obstetrical bleeding).^20^ The ESRAT consists of 330 questions regarding infrastructure, staffing, staff professional development, medications, laboratory studies, and equipment. The Spanish version of the ESRAT uses the term *sala de emergencias* [emergency room], which in practice refers to any physical space in which emergencies are treated. As the WHO uses the term “emergency unit” (EU),^21^ we have done the same in this paper. We modified the tool for the local context through an extensive pilot survey of the first participating facility, and the resultant modified tool was used for subsequent surveys. Of the 41 modifications to the survey tool, 33 (80%) were differences in translation due to the unique vocabulary used by the local population. The survey tool also specified whether laboratory and/or blood bank services were available in-house or via contract with an external vendor. Additionally, the tool was used to specifically ask whether there was a dedicated EU. Finally, we removed one laboratory study and two medications as malaria is not endemic in the studied region, nor is it home to venomous snakes that would pose a risk of snake bites.

### Analysis

We summarized infrastructure and staffing in narrative form due to the heterogenous nature of the various facilities with regard to these categories. Performance was quantified for equipment, labs, and medications and given as a percentage of the number of observed items in that category, divided by the total number of items in that category in ESRAT. We assigned a total score as a percentage of total possible points, and points were assigned to each survey item response as specified in the survey tool. The total score category was designed to capture the survey’s multiple small categories, which would otherwise be difficult to report individually; included in this category, for example, would be whether a facility required patients to pay prior to receiving care or whether a facility had quality improvement protocols.

We grouped studied facilities by facility level and funding source with the three resultant groups: private hospital; public hospital; and public clinic. Hospitals were analyzed separately from clinics due to hypothesized differences in resource availability. We analyzed public and private hospitals separately due to the known underfunding of public healthcare in Guatemala^22^ and thus hypothesized a lower level of resources. No private clinics were identified that provided urgent or emergent care. Facilities were then grouped by city in which they were located to ascertain whether there was a difference in level of available care between cities.

Data were entered into Microsoft Excel version 2006 (Microsoft Corporation, Redmond, WA). We used a 3×4 factorial ANOVA with Tukey’s honest significant difference test to compare group means between facility types and cities for equipment, labs, medications, and total score. All subgroup analyses were defined a priori. There were no missing data. Adjusted *P*-values are reported to account for multiple comparisons. An alpha value of 0.05 was considered significant. We performed data analysis and visualization in R version 4.0.1 (R Foundation for Statistical Computing, Vienna, Austria).

### Ethics

This study was reviewed and determined exempt by the institutional review board of the University of Wisconsin. This research did not receive any specific grant from funding agencies in the public, commercial, or not-for-profit sectors.

## RESULTS

### Characteristics of Study Subjects

We identified 17 facilities in the target region, of which 16 agreed to participate. Among the 16, there were nine private hospitals, four public clinics, and three public hospitals, including the region’s public departmental hospital located in Sololá (Table).

### Infrastructure

All facilities provided emergency services 24/7, and a dedicated EU was available at 67% of hospitals and 75% of clinics. Access to consistent electricity and running water was near universal, with one clinic reporting only “sometimes” having running water instead of “always.” In addition to emergency services, almost all hospitals offered ambulatory (100%), surgical (92%), and pharmacy (100%) services, whereas only about half offered blood bank (58%) and radiologic services (58%). Among clinics, only pharmacy was a consistent service (75%).

### Staffing

A general physician was assigned to every facility and on call 24/7 for the entire facility. In addition, most hospitals had an anesthesiologist (75%), an obstetrician (83%), and a surgeon (83%) on staff. Only 33% of hospitals had a radiologist on staff. No clinic had any specialist physicians. A dedicated physician was in the EU during the day at 67% of hospitals and 75% of clinics. When a physician was not present, a registered nurse or nurse assistant was in the unit. After hours, every hospital and 75% of clinics had a physician in-house or on call 24/7, specifically for the EU.

### Equipment

**Figure 1:**
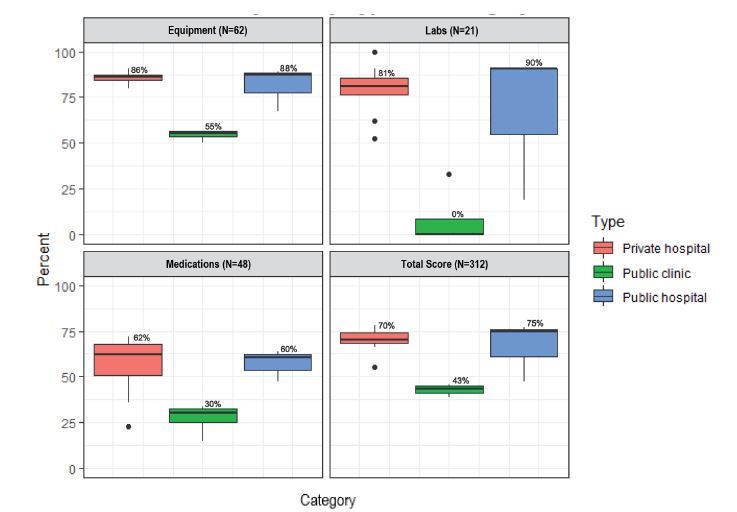
Performance by facility type and category. The displayed values are as follows: whiskers - 1.5* interquartile range; hinges - 25th and 75th percentile; middle - median.

In general, availability of equipment was high in all facilities (Figure 1). Access to personal protective equipment (PPE), including masks and non-sterile gloves as well as basic wound care supplies, was universal among facilities. Among clinics, the largest deficit was in airway equipment (0–50% depending on item) and trauma equipment such as C-collars (0%), splints (0%), and large-bore intravenous (IV) needles (18G or larger, 75%). Among hospitals, the largest deficit was in pediatric equipment such as C-collar (8%), blood pressure cuff (83%), and intubation equipment (75–83%, depending on item). Availability of larger equipment—such as electrocardiogram machine, ultrasound machine (point of care or comprehensive), and suction machine—was more variable among hospitals and almost non-existent among clinics. Eleven of 16 facilities had at least 70% of surveyed equipment. Hospitals had a significantly higher percentage of available equipment compared to clinics (85% vs 54%, mean difference 31%; 95% confidence interval (CI) 23–37%; *P* = 0.004). There was no difference in equipment availability between public and private hospitals (*P* = 0.57) or between cities (*P* = 0.80).

### Laboratory Tests

Availability of lab tests was generally high among hospitals but very low among clinics (Figure 1, 76% vs 8%, mean difference 67%, 95% CI 41–93%; *P* = 0.004). The one clinic with any laboratory services only offered basic point-of-care labs. Among hospital laboratories, most labs were available at every facility. The largest shortcomings were regarding bacterial cultures, as well as anything related to cerebrospinal fluid (including microscopy, basic studies, and culture). There was no difference in laboratory test availability between public and private hospitals (*P* = 0.66) or between cities (*P* = 0.43).

### Medications

The weakest measure among a facility’s physical assets was medications, even for hospitals (Figure 1). Only a quarter of facilities checked medication stocks daily, although every facility reported appropriate storage and refrigeration of medications. Hospitals had good access to oxygen (100%), inhaled bronchodilators (100%), IV fluids (100%), and antibiotics (25–83%, depending on item) vs clinic availability of 25%, 50%, 100%, and 0–100%, respectively. Epinephrine and regular insulin were not always available (mean of 92% and 67% for hospitals and 50% and 25% for clinics, respectively). Only two of 16 facilities had at least 70% of surveyed medications. Private hospitals had access to a significantly higher percentage of medications compared to clinics (56% vs 27%, mean difference 29%; 95% CI 9–49%; *P* = 0.024). However, there was no difference in medication access between clinics and public hospitals (*P* = 0.06), public and private hospitals (*P* = 0.99), or between cities (*P* = 0.634).

### Total Score

Given the large number and heterogenous nature of survey tool items, the total score category was created to allow comparison of these multiple smaller categories. We report here a subset of these items. (For a full list see the tool in Supplemental Materials.) An ambulance was available at all hospitals but at only 50% of clinics. Of the facilities with an ambulance, hospitals’ ambulances had a mean of 37% of surveyed equipment, while clinics’ ambulances had a mean of 23%. All public facilities had triage protocols for the EU, compared to only 67% of private hospitals. The average frequency of professional training opportunities among all facilities was 9.6 trainings per year for both nurses and physicians. Twenty-two percent of private hospitals required payment before providing services, even in cases of emergency, although no public facility reported this practice. Only 63% of facilities had a mass casualty plan, but none had practiced the plan within the prior year. Hospitals had a greater availability of all survey items (total score) than clinics (69% vs 43%, mean difference 26%; 95% CI 15–36%; *P* = 0.008). There was no difference in total score between public and private hospitals (*P* = 0.82), or between cities (*P* = 0.51).

## DISCUSSION

This study describes the regional, self-reported availability of emergency services in a rural area of Guatemala serving a predominantly indigenous population. This is the first systematic assessment of capacity in rural Central and South America. Previous work was limited to one study of emergency services in an urban area in El Salvador^10^ and other studies in South America with limited scopes of assessment such as trauma or surgery.^11^–^15^ In our study we found some areas of adequate capacity including in supplies such as PPE and staffing infrastructure. However, there were also major deficiencies including a severe shortage of critical medications in clinics, and a widespread shortage of pediatric equipment.

In terms of positive findings, we found high availability of basic PPE in all surveyed facilities, similar to conditions in Myanmar^23^ and Ghana.^24^ It should be noted, however, that our study was conducted before the coronavirus 2019 pandemic and may not reflect global changes and shortages of PPE.^25^ Our survey also reflected broadly high availability of general emergency supplies such as airway equipment, IV equipment, and vitals monitoring equipment. This finding is similar to others in the region including a survey of facilities in El Salvador 10 and in other areas of the globe including surveys of hospitals in Kenya,^26^ Sierra Leon,^27^ and Zambia.^28^

The access to a physician in the EU was surprisingly high, in contrast to other studies where after-hours access was as low as 38% of facilities.^10^ However, in our population there were no emergency medicine (EM)-trained physicians available at any time. While there are no studies comparing the outcomes of EM board-certified physicians to general practitioner physicians, the three major US EM professional societies have policy statements regarding the superior care from an EM board-certified physician in an emergency setting.^29^–^31^ However, access to an EM-trained physician is limited in Guatemala, as there are only two EM residencies in the country, the first of which was founded in 2017, and both of which are based in the capital Guatemala City.^32^

We found a significant shortcoming in the availability of critical medications such as oxygen and epinephrine in surveyed clinics, and even hospitals had barely greater than half of medications available. This is compared to 60% medication availability in El Salvador.^10^ A similar study in urban and rural Myanmar found universal availability of oxygen and oral antibiotics at all facility levels,^23^ although in that study and all others, the availability of epinephrine was not assessed in clinics.^23^,^24^,^27^,^28^,^33^

Like the limitations in medication and oxygen, we also found a significant deficiency in pediatric equipment availability across all facilities. Previous studies have not conducted comprehensive pediatric emergency assessments, and this is the first report to do so. Pediatric emergency services are often cited as an area in need of improvement both in LMIC countries^34^ and high-resource countries such as the US.^35^ Our recommendation for improvement in this area is to increase the priority of pediatric supplies when making funding decisions.

Significant work in assessing causes of stockouts in the Guatemalan healthcare system has been undertaken by the US Agency for International Development, and its most recent Health Systems Assessment^19^ has several pertinent recommendations, which we would echo. While the need for increased funding is a constant refrain, other interventions would be to automate inventory management, focus on providing medications and supplies that are actually being used, and focus on stocking a smaller number of core medications and supplies. Reassuringly, we found no significant variation between availability of services between private and public facilities or between cities in this region, in contrast to the inferiority of public facilities reported in Sierra Leone.^27^

Although the ESRAT has been used in a variety of LMICs, it has not been formally validated. However, it is similar to other emergency systems survey tools such as the Emergency Care Assessment Tool^36^ and the Hospital Emergency Unit Assessment Tool^33^ and covers every clinical category in those surveys (termed “signal functions” by those tools) with the addition of obstetrical bleeding.

The major findings of our assessment were a high availability of emergency services, universal PPE availability, a severe shortage of critical medications in clinics, and widespread shortage of pediatric equipment. Medication availability was the largest area of need, as only two facilities met the 70% target set by the WHO for supply of basic emergency medications.^24^ Equipment and supplies fared much better, with 11 of 16 facilities meeting the 70% target. Although many clinic settings reported providing acute care services 24/7, there was limited availability of personnel, medications, and equipment. Both public and private hospitals reported similar capabilities.

Although there are a number of settings where residents of the Atitlán area can access emergency services, the actual access of and use by residents of the region to these facilities is not clear. There is no prehospital system within this area (and it is still limited in much of Guatemala),^9^ so that patients self-triage to various facilities. Understanding the factors that lead patients to use one facility over another — geography,^37^,^38^ expense,^39^,^40^ or perceived acuity — is important for further defining emergency services within the area.

The flow of patients within this system from one facility to another is also unclear, including whether some of these facilities function mostly as triage to the larger hospitals or provide definitive care. Although the ESRAT was not designed to assess clinics, we included these facilities in this assessment because they reported providing emergency services, and public clinics are often the only option for people with limited financial means as the only public hospitals in the region are located in Sololá and Panajachel, which both require a lengthy boat or car ride to be reached from other studied areas. Additionally, due to the mountainous setting, roads may be impassable following natural disasters, and patients may not be able to reach a hospital. Indeed, in the immediate aftermath of Hurricane Stan in 2005, it was reported in a community in Sololá’s neighboring department that 61% of the population was unable to get to a hospital for injuries sustained from the hurricane.^41^ Thus, it was essential to assess and improve the ability of clinics to provide basic management and stabilization before transfer as patients are presenting to them with emergency needs. Notably, access to basic life-saving capabilities such as oxygen was very low in these clinics.

## LIMITATIONS

While we believe we identified all facilities providing emergency services in the area, there may be facilities that were missed, and one facility did not agree to participate in our study. The facility that declined to participate was a private hospital in Sololá which, based on public marketing materials and preliminary conversations with hospital leadership, provided services similar to the other private hospitals surveyed.

Some results of the survey are self-reported and thus limited by personal knowledge of the respondents. This was in part mitigated by interviewing more than one person at each facility, performing all counts of resources (infrastructure, supplies, and medications) by direct inspection, and directly visualizing staffing rosters. Another limitation is that the ESRAT evaluates only the presence of items and not personnel trained in their use. Thus, our findings are likely an overestimation of each facility’s true capacity. Finally, it should be noted that the survey used was not originally developed in Spanish; therefore, some errors in translation and transcription may be present, although these were mitigated by the survey optimization process and the use of a single bilingual surveyor.

These results address only one rural area in Guatemala, which limits generalizability. It is unclear whether the deficiencies identified in this area are universal to all regions in Guatemala. We suspect that they may in fact be more pronounced in this region given the largely indigenous population and known disparities that exist in the healthcare system between urban and rural areas.^42^ Because Guatemala does have a national healthcare system, it is possible that the availability of resources would be similar in public healthcare facilities across the country.

## CONCLUSION

We found that emergency units serving a rural, largely indigenous population in Guatemala demonstrated several critical deficiencies, most prominently in medications and pediatric-specific equipment. There were also large discrepancies between hospitals and clinics, such as availability of specialists and laboratory services, While such discrepancies may be expected, they also pose challenges for patients who do not know or understand these variations. As emergency services develop across Central and South America, it is important to understand the critical shortages facing these facilities, especially in rural areas. Future studies in acute care use and patient outcomes are needed to better understand how to improve emergency services for rural populations.

## Supplementary Information



## Figures and Tables

**Table:** Number of facilities of each type in the four studied Guatemalan cities.

City	Private hospitals (N)	Public hospitals (N)	Public Clinics (N)
Sololá	2	2	1
Panajachel	3	1	0
Santiago	2	0	1
San Lucas Tolimán	2	0	2
