# Supplementary material for: Emergency Services Capacity of a Rural Community in Guatemala
Source: West J Emerg Med. 2022 Sep 12;23(5):746–53. doi: 10.5811/westjem.2022.7.56258 (PMC9541976; doi:10.5811/westjem.2022.7.56258)
Supplement: Supplementary file 1 [file wjem-23-746-s001.pdf]

Herramienta de Evaluación de Necesidades SidHARTe

sidHARTe
Strengthening Emergency Systems

PESTAÑA 1: ADMINISTRADOR

Nombre Entrevistador ______________________________ Fecha (dd/mm/aa): ___ / ___ / ___

1. SERVICIOS PRESTADOS Y HORARIO DE ATENCIÓN

INSTRUCCIONES: Estas preguntas deben ser dirigidas al administrador senior del hospital. Verifique la existencia de cada departamento cuando evalúe el hospital.

1.1	Nos gustaría saber acerca de algunos de los servicios básicos que se prestan en esta instalación. La instalación ofrece (lea cada ítem):	Sí	No
	a. ¿Clínica externa?	1	0
	b. ¿Atención de emergencia?	1	0
	b1.¿Sala de emergencia separada?	1	0
	c. ¿Atención quirúrgica (procedimientos de cirugía general básicos: apendicetomía, reparaciones de perforación por fiebre tifoidea, reparaciones de hernia)?	1	0
	d. ¿Servicios de farmacia?	1	0
	e. ¿Servicios de laboratorio en el sitio?	1	0
	e1. ¿Servicios de laboratorio de un servicio contratado?	1	0
	f. ¿Banco de sangre en el sitio?	1	0
	f1. ¿Banco de sangre de un servicio contratado?	1	0
	g. ¿Servicio de Rayos X?	1	0
	h. ¿Su propia ambulancia?	1	0
	h1. ¿Ambulancia de un servicio contratado?	1	0
1.2	¿La sala de emergencias está abierta 24 horas/día y 7 días/semana?	Sí..1<br>No 0	

PESTAÑA 2: OFICIAL RR. HH.

Nombre Entrevistador ______________________________ Fecha (dd/mm/aa): ___ / ___ / ___

INSTRUCCIONES: Estas preguntas deben ser dirigidas al jefede recursos humanos del hospital.

1. USO Y MANTENIMIENTO ADECUADO DE EQUIPOS

1.1	La siguiente pregunta tiene que ver con el uso adecuado de ciertas piezas de equipo en el hospital: ¿El hospital cuenta con un miembro del personal capacitado en ultrasonido disponible en las instalaciones las 24 horas?	Sí …………….……………1<br>No………….………………0

2. PERSONAL DE HOSPITAL EN EJERCICIO

INSTRUCCIONES: Haga la pregunta 2.1, luego baje por la lista de personal del hospital. Repita con la pregunta 2.2. Confirmar con listas de personal.

		Sí	No
2.1	La siguiente pregunta averigua sobre personal de distintos grados en este hospital. ¿Puede mostrarme sus listas de personal? Algunos de estas dotaciones de personal están asignadas o publicadas en el hospital (leer cada una de ellas):		
	a1. Anestesiólogo	1	0
	a2. Radiólogo	1	0
	a3. Obstetra	1	0
	a4. Cualquier cirujano	1	0
	a5. Generalista	1	0
	b. Asistentes médicos/otros prescriptores	1	0
	c1. Enfermeras de cuidados críticos	1	0
	c2. Anestesistas enfermeros	1	0
	c3. Partera/comadrona/comadre	1	0
	c4. Enfermeras generales	1	0
	c5. Enfermeras auxiliares/profesional clínico asistente de salud	1	0
	d. Trabajadores de extensión de salud	1	0
	e. Farmacéuticos/Técnicos Farmacéuticos/Técnicos de Dispensario/Tecnólogos	1	0
	f. Científicos biomédicos	1	0

	g. Asistentes/técnicos/tecnólogos de laboratorio	1	0
	h. Radiólogo/Técnicos de rayos X	1	0
	i. Asistentes de sala/camilleros	1	0
2.2	¿Alguno de estos miembros del personal están asignados para responder a llamadas o estar disponibles 24 horas (lea cada ítem):	Sí	No
	a1. Anestesiólogo	1	0
	a2. Radiólogo	1	0
	a3. Obstetra	1	0
	a4. Cualquier cirujano	1	0
	a5. Generalista	1	0
	b. Asistentes médicos/otros prescriptores	1	0
	c1. Enfermeras de cuidados críticos	1	0
	c2. Anestesistas enfermeros	1	0
	c3. Partera/comadrona/comadre	1	0
	c4. Enfermeras generales	1	0
	c5. Enfermeras auxiliares/profesional clínico asistente de salud	1	0
	d. Trabajadores de extensión de salud	1	0
	e. Farmacéuticos/Técnicos Farmacéuticos/Técnicos de Dispensario/Tecnólogos	1	0
	f. Científicos biomédicos	1	0
	g. Asistentes/técnicos/tecnólogos de laboratorio	1	0
	h. Radiólogo/Técnicos de rayos X	1	0
	i. Asistentes de sala/camilleros	1	0

3. PERSONAL DE SALA DE EMERGENCIA (O SEA, PERSONAL PERMANENTE EN SALA DE EMERGENCIA)

INSTRUCCIONES: Haga la pregunta 3.1, luego baje por la lista de personal del hospital. Repita con la pregunta 3.2. Confirmar con listas de personal.

3.1	Las siguientes preguntas averiguan el número de miembros de personal de diversos grados en la sala de emergencia. ¿Puede mostrarme sus listas de personal? Algunos de estas dotaciones de personal están asignadas o publicadas en la Sala de Emergencias (lea cada ítem):	Sí	No
	a1. Médico capacitado para atención de emergencias	1	0
	a2. Otro médico	1	0
	b. Asistentes médicos/otros prescriptores	1	0
	c1. Enfermera capacitada para atender emergencias	1	0
	c2. Enfermeras de cuidados críticos	1	0
	c3. Enfermeras generales	1	0
	d. Trabajadores de extensión de salud	1	0
	e. Asistentes de sala/camilleros	1	0
3.2	¿Alguno de estos miembros del personal están asignados para responder a llamadas o estar disponibles 24 horas en la Sala de Emergencia (lea cada ítem):	Sí	No
	a1. Médico capacitado para atención de emergencias	1	0
	a2. Otro médico	1	0
	b. Asistentes médicos/otros prescriptores	1	0
	c1. Enfermera capacitada para atender emergencias	1	0
	c2. Enfermeras de cuidados críticos	1	0
	c3. Enfermeras generales	1	0
	d. Trabajadores de extensión de salud	1	0
	e. Asistentes de sala/camilleros	1	0

4. CAPACITACIÓN

4.1	La siguiente pregunta averigua sobre oportunidades de capacitación profesional para personal del hospital: En los últimos 3 meses, ¿cuántas veces se han ofrecido oportunidades de capacitación/educación continua en Medicina en el lugar para (lea opciones)?	a. ¿facultativos/asistentes médicos/prescriptores? __________<br>b. ¿Enfermeras? __________

5. MOTIVACIÓN

5.1	Las siguientes preguntas averiguan sobre maneras en que el hospital puede elegir para motivar al personal del hospital: ¿El hospital o la sala de emergencia tiene algún sistema formal para reconocimiento público de rendimiento excelente del personal?	Sí .... 1<br>No 0
5.2	¿El hospital ofrece ascensos o incrementos de salario sobre la base de rendimiento excelente?	Sí .... 1<br>No 0
5.3	¿El hospital aboga por ascensos o incrementos de salario por parte del gobierno sobre la base de rendimiento excelente?	Sí .... 1<br>No 0
5.4	¿El hospital brinda apoyo financiero a aquellos miembros del personal que quieren continuar su educación?	Sí .... 1<br>No 0
5.5	¿El hospital ofrece horario de trabajo flexible a aquellos miembros del personal que quieren continuar su educación?	Sí .... 1<br>No 0

6. CAPACIDAD Y CAPACITACIÓN DEL CONDUCTOR

6.1	La siguiente pregunta averigua sobre la capacitación del conductor de ambulancia: ¿El conductor(es) ha (n) recibido alguna capacitación en primeros auxilios?	Sí .... 1<br>No 0

7. CAPACITACIÓN/CALIFICACIÓN DE PERSONAL DE ATENCIÓN EN TRIAJE

7.1	La siguiente pregunta averigua sobre la capacitación para el protocolo de triaje del hospital: ¿Tanto enfermeras como prescriptores (Doctores en Medicina/Asistentes Médicos) están capacitados en el uso del protocolo de triaje?	Sí .... 1<br>No 0

PESTAÑA 3: RESPONSABLE DE SALA DE EMERGENCIA

Nombre Entrevistador ______________________________ Fecha (dd/mm/aa): ___ / ___ / ___

INSTRUCCIONES: Estas preguntas deben ser dirigidas al responsable de sala de emergencia.

1. SERVICIOS PRESTADOS Y HORARIO DE ATENCIÓN

1.1	La siguiente pregunta averigua sobre los servicios que este hospital ofrece y el horario de atención de los diversos departamentos: ¿Los siguientes departamentos respondieron a su última llamada fuera de horario de atención normal? (lea cada ítem):	Sí	No
	a. la clínica externa	1	0
	b. la sala de cirugías	1	0
	c. la farmacia	1	0
	d. el laboratorio	1	0
	e. el banco de sangre	1	0
	f. el departamento de rayos x	1	0

2. SERVICIOS DE ATENCIÓN DE EMERGENCIA

2.1	Las siguientes preguntas averiguan sobre los servicios de atención de emergencia que la clínica externa ofrece en este hospital: En los últimos 7 días, ¿la Sala de Emergencias ha recibido pacientes que pudieron haber sido atendidos por la clínica externa cuando seguía abierto?	Sí.................................0<br>No................................ 1
2.2	En los últimos 7 días, ¿la Sala de Emergencias ha recibido pacientes que pudieron haber sido atendidos la clínica externa al día siguiente en que estuvo cerrado?	Sí................................0<br>No............................... 1

3. BANCO DE SANGRE

INSTRUCCIÓN: Si el hospital no tiene un banco de sangre, pase directamente a la SECCIÓN 4.

3.1	La siguiente pregunta averigua sobre la disponibilidad de sangre en el banco de sangre de este hospital: En situaciones de emergencia, ¿con qué frecuencia la sala de emergencia recibe sangre del banco de sangre del hospital? (lea opciones)	Todo el tiempo ....2<br>A veces.................................1<br>Nunca 0

4. ELECTRICIDAD Y AGUA

4.1	Las siguientes preguntas averiguan sobre el suministro de electricidad y agua en este hospital: ¿Con qué frecuencia hay electricidad disponible en la sala de emergencia? (lea opciones)	Todo el tiempo ....2<br>A veces..................................1<br>Nunca.................................... 0
4.2	¿Con qué frecuencia hay agua corriente disponible en la sala de emergencia? (lea opciones)	Todo el tiempo ....2<br>A veces..................................1<br>Nunca.................................... 0
4.3	La última vez que el suministro de agua local se interrumpió, ¿se proveyó de agua desde otra fuente?	Sí ....1<br>No.. 0

5. SISTEMA DE PAGO/BARRERAS FINANCIERAS PARA LA ATENCIÓN

5.1	Las siguientes preguntas averiguan sobre cómo se administra la carga financiera de la atención de emergencia en este hospital:<br>¿Alguna vez se demora la atención de emergencia para pacientes asegurados debido a que ellos deben hacer un pago inicial?	Sí ....0<br>No.. 1
5.2	¿Alguna vez se demora la atención de emergencia para pacientes no asegurados debido a que ellos deben hacer un pago inicial?	Sí ....0<br>No.. 1

6. ASEGURAMIENTO DE LA CALIDAD

		Sí	No
6.1	La siguiente pregunta averigua sobre las medidas de aseguramiento de la calidad utilizadas por este hospital: Hasta donde usted sabe, ¿la sala de emergencia hace lo siguiente como parte de sus medidas de aseguramiento de la calidad? (lea cada ítem):		
	a. ¿auditorías clínicas (morbilidad y mortalidad o revisiones de casos)?	1	0
	b. ¿documentación estandarizada (formato estándar para anotaciones de pacientes, preguntas de consumo estándar) en historia clínica de pacientes?	1	0
	c. ¿reportes de eventos adversos?	1	0
	d. ¿medidas de control de infecciones estandarizadas?	1	0
	e. ¿uso de protocolos o pautas clínicas para pacientes de emergencia?	1	0

	f. ¿educación continua en Medicina o capacitaciones en servicio?	1	0
	g. ¿supervisión clínica?	1	0

7. ALMACENAMIENTO Y REPOSICIÓN DE MEDICAMENTOS

INSTRUCCIÓN: Coteje las siguientes respuestas con la situación real de los medicamentos que se mantienen en la sala de emergencia

7.1	Las siguientes preguntas averiguan sobre la accesibilidad de medicinas para emergencia en este hospital: ¿Tiene la sala de emergencia su propia existencia de medicamentos para emergencia?	Sí..1<br>No 0
7.2	¿Los medicamentos de sala de emergencia están protegidos de humedad, calor o infestación (p. ej., puestos en repisas, ventilados)?	Sí..1<br>No 0
7.3	¿Efectivamente todos los proveedores de atención de emergencia tienen acceso a la existencia de medicina de emergencia?	Sí..1<br>No 0
7.4	¿Los medicamentos de sala de emergencia que lo requieren están almacenados refrigerados en un refrigerador que funciona?	Sí..1<br>No 0
7.5	¿Con qué frecuencia el personal de sala de emergencia verifica las cantidades de medicamentos de emergencia esenciales?	Al menos una vez al día.........1<br>Menos de una vez al día........0

8. USO Y MANTENIMIENTO ADECUADO DE EQUIPOS

8.1	Las siguientes preguntas averiguan sobre el uso y el mantenimiento de equipos en este hospital: ¿Está usted en condiciones de contactarse con el miembro del personal capacitado en ultrasonido para realizar escáneres de emergencia fuera de horario de atención normal?	Sí..1<br>No.. 0

8.2	¿La orientación del personal nuevo incluye instrucción sobre cómo usar lo siguiente? (lea cada ítem):	Sí	No
	a. máquina de succión/aspiradora	1	0
	b. máquina nebulizadora	1	0
	c. concentrador de oxígeno	1	0
	d. monitor cardiopulmonar	1	0
8.3	¿Hay algún insumo o equipo de sala de emergencia guardado bajo llave?	Sí ....1<br>No 0	
8.4	¿El miembro de personal primario que trabaja en sala de emergencia siempre tiene la llave?	Sí ....1<br>No 0	
8.5	¿Usted ve que se realice un mantenimiento rutinario de lo siguiente? (lea cada ítem):	Sí	No
	a. máquina de succión/aspiradora (sala de emergencia)	1	0
	b. máquina nebulizadora (sala de emergencia)	1	0
	c. concentrador de oxígeno (sala de emergencia)	1	0
	d. dispositivo de monitoreo cardiopulmonar (sala de emergencia)	1	0
8.6	¿El hospital repara y/o reemplaza equipo pequeño (esfigmomanómetros, estetoscopios, etc.) según sea necesario?	Sí ....1<br>No 0	

9. CAPACITACIÓN

9.1	Las siguientes preguntas averiguan sobre oportunidades de capacitación profesional para personal del hospital: ¿Se imparte capacitación en servicio en sala de emergencia, con personal superior enseñando a personal junior mientras ellos atienden pacientes?	Sí ....1<br>No 0

INSTRUCCIÓN: Si la respuesta a esta pregunta es NO, DESCONOCE, o NO APLICA, favor pasar directamente a la SECCIÓN 10.

9.2	Si es así, ¿con qué frecuencia ocurre la enseñanza en servicio?	Una vez al mes o más .... 1<br>Menos de una vez al mes............0

10. SUPERVISIÓN

10.1	Las siguientes preguntas averiguan sobre la supervisión del personal del hospital: ¿Con qué frecuencia usted normalmente hace que un supervisor clínico (Matrona Enfermera, Facultativo/Prescriptor, o Encargado de Sala de Emergencia) hable con usted acerca de su trabajo o bien que observe su trabajo?	Una vez al mes o más .... 1<br>Menos de una vez al mes 0
10.2	En la última ocasión en que su supervisor observó el trabajo de usted, ¿evaluó él/ella su documentación en los historiales de pacientes?	Sí .... 1<br>No 0
10.3	En la última ocasión en que su supervisor observó el trabajo de usted, ¿evaluó él/ella sus habilidades para exámenes físicos, habilidades para procedimientos, u otras habilidades clínicas?	Sí .... 1<br>No 0

11. DISPONIBILIDAD DE TRANSPORTE/EQUIPO/MANTENIMIENTO

11.1	Las siguientes preguntas averiguan sobre el vehículo(s) de transporte y las prácticas de transporte para referir pacientes a atención de mayor nivel: ¿Con qué frecuencia está disponible el vehículo para transporte de emergencia primaria?	Siempre .... 2<br>A veces................................ 1<br>Nunca 0
11.2	En los últimos tres meses, ¿ha experimentado alguna demora en transferencias de pacientes debido a que la ambulancia estaba fuera de servicio?	Sí .... 1<br>No 0
11.3	¿Alguna vez ocurre que las familias deciden no transferir a un paciente debido al costo del transporte?	Siempre .... 2<br>A veces................................ 1<br>Nunca 0

12. DISPONIBILIDAD DEL CONDUCTOR

12.1	La siguiente pregunta averigua sobre la disponibilidad del conductor de ambulancia: ¿Con qué frecuencia hay un conductor disponible?	Siempre .... 2<br>A veces................................ 1<br>Nunca 0

13. CALIFICACIONES DEL PROVEEDOR ACOMPAÑANTE

13.1	La siguiente pregunta averigua sobre el proveedor de atención de salud que acompaña a los pacientes que son referidos a otra instalación: ¿Con qué frecuencia hay disponible un proveedor de atención de salud capacitado (sin incluir al conductor) para acompañar a los pacientes?	Siempre .... 2<br>A veces................................ 1<br>Nunca 0

14. COMUNICACIONES PARA FINES DE REFERENCIA

INSTRUCCIÓN: Coteje las siguientes respuestas con la presencia real de equipo de comunicaciones, lista de hospitales de referencia, y formulario de reporte para transferencia en la sala de emergencia.

14.1	Las siguientes preguntas averiguan sobre el sistema de comunicación utilizado para referencias: ¿La sala de emergencia tiene un teléfono de línea fija o un teléfono móvil operativo?	Sí...................................... 1<br>No 0	
14.2	¿Se llama a la siguiente persona cuando un paciente necesita ser transferido si ellos no están ya presentes? (lea cada ítem):	Sí	No
	a. proveedor de atención de salud acompañante	1	0
	b. Doctor en Medicina/Asistente Médico de turno en hospital de referencia	1	0
	c. conductor de ambulancia	1	0
	d. familia del paciente	1	0
14.3	¿Hay una lista de hospitales de referencia con números de teléfono publicada en la sala de emergencia?	Sí.................................. 1<br>No.................................. 0	

15. ORGANIZACIÓN DE SALA DE EMERGENCIA PARA TRIAJE

INSTRUCCIÓN: Coteje la siguiente respuesta con la presencia real de organización de triaje en la sala de emergencia.

15.1	La siguiente pregunta averigua sobre la organización de la sala de emergencia a lo largo de categorías de triaje: ¿La organización de la sala de emergencia refleja el protocolo de triaje de alguna manera (es decir, con brazaletes codificados por color, camas, historiales clínicos o áreas)?	Sí .... 1<br>No 0

PESTAÑA 4: RESPONSABLE DE DEPARTAMENTO DE ATENCIÓN AMBULATORIA

Nombre Entrevistador ______________________________ Fecha (dd/mm/aa): ___ / ___ / ___

INSTRUCCIONES: Estas preguntas deben ser dirigidas al prescriptor o enfermera responsable que dirija la clínica externa.

1. SERVICIOS DE ATENCIÓN DE EMERGENCIA

INSTRUCCIONES: Coteje las siguientes respuestas con la presencia de equipo real en la consulta externa.

		Sí	No
1.1	Las siguientes preguntas averiguan sobre los servicios de atención de emergencia que la clínica externa ofrece en este hospital: ¿La clínica externa tiene el equipo y los insumos para ofrecer las siguientes intervenciones? (lea cada ítem)		
	a. ¿medicamentos nebulizados?	1	0
	b. ¿medicamentos IV o IM?	1	0
	c. ¿atención de heridas básicas?	1	0

PESTAÑA 5: BANCO DE SANGRE y LABORATORIO

Nombre Entrevistador ______________________________ Fecha (dd/mm/aa): ___ / ___ / ___

INSTRUCCIONES: Estas preguntas deben ser dirigidas al técnico de laboratorio o científico biomédico que dirija el laboratorio y el banco de sangre.

1. BANCO DE SANGRE

INSTRUCCIONES: Coteje las siguientes respuestas con la presencia de productos sanguíneos reales en el banco de sangre.

1.1	Las siguientes preguntas averiguan sobre el banco de sangre del hospital: ¿El laboratorio ofrece acceso las 24 horas para prueba de compatibilidad sanguínea?	Sí .... 1<br>No 0
1.2	En el último mes, ¿con qué frecuencia hubo disponibilidad de transfusiones sanguíneas en el hospital?	Siempre .... 2<br>A veces................................. 1<br>Nunca 0

2. LABORATORIO

		Sí	No
2.1	Las siguientes preguntas averiguan sobre el laboratorio del hospital: ¿El laboratorio ofrece los siguientes pruebas o servicios: (lea cada ítem)		
	a. ¿tinción de Gram?	1	0
	b. ¿tinción acidorresistente?	1	0
	c. ¿nivel de hemoglobina?	1	0
	d. ¿conteo de glóbulos blancos?	1	0
	e. ¿Tipificación y compatibilidad sanguínea?	1	0
	f. ¿Frotis de sangre para parásitos de malaria?	1	0
	g. ¿Nivel de glucosa en la sangre?	1	0
	h. ¿Pruebas de VIH?	1	0
	i. ¿Examen microscópico fluido cerebroespinal?	1	0
	j. ¿Examen microscópico orina?	1	0
	k. ¿Examen microscópico heces?	1	0
	l. ¿Panel electrolítico?	1	0
	m. Estudios de coagulación (CUALQUIERA DE LOS SIGUIENTES: TTPa/tiempo protrombina/INR/tiempo de coagulación)?	1	0
	n. Estudios de fluido cerebroespinal (CUALQUIERA DE LOS SIGUIENTES: glucosa/proteína/etc.)?	1	0
	o. ¿prueba de función hepática?	1	0
	p. Estudios renales (CUALQUIERA DE LOS SIGUIENTES: BUE/Creatinina)?	1	0
	q. Marcadores cardiacos (CUALQUIERA DE LOS SIGUIENTES: troponinas/CK-MB/LDH/AST)?	1	0
	r. ¿Cultivos sanguíneos?	1	0
	s. ¿Cultivos de fluido cerebroespinal?	1	0
	t. ¿Cultivos de orina?	1	0
	u. ¿Cultivos de heces?	1	0

PESTAÑA 6: CONTABILIDAD

Nombre Entrevistador ______________________________ Fecha (dd/mm/aa): ___ / ___ / ___

INSTRUCCIONES: Estas preguntas deben ser dirigidas al jefe de cuentas del hospital.

1. SISTEMA DE PAGO/BARRERAS FINANCIERAS PARA LA ATENCIÓN

1.1	Las siguientes preguntas averiguan sobre los sistemas de pago para pacientes de emergencia en este hospital: ¿Existe un sistema formal vigente para que pacientes pobres queden exentos de pagar las tarifas por servicios de emergencia?	Sí.................................1<br>No.................................0
1.2	¿El hospital tiene un sistema para permitir a pacientes pobres pagar después?	Sí.................................1<br>No.................................0

2. MOTIVACIÓN

2.1	Las siguientes preguntas averiguan sobre salarios y otros incentivos financieros para miembros del personal en este hospital: ¿El hospital tiene un calendario regular para el pago de salarios a trabajadores casuales?	Sí.................................1<br>No.................................0
2.2	¿El hospital tiene un calendario regular/día de pago para el pago de salarios al personal permanente?	Sí.................................1<br>No.................................0
2.3	¿Algún empleado aún no ha recibido su salario correspondiente al día de pago más reciente?	Sí.................................0<br>No.................................1
2.4	¿El hospital ofrece alguna vivienda a trabajadores empleados por el gobierno?	Sí.................................1<br>No.................................0
2.5	¿El hospital ofrece algún pago de salario adicional a trabajadores empleados por el gobierno?	Sí.................................1<br>No.................................0
2.6	¿El hospital ofrece algún préstamo financiero a trabajadores empleados por el gobierno?	Sí.................................1<br>No.................................0

3. DISPONIBILIDAD DE TRANSPORTE/MANTENIMIENTO/EQUIPO

3.1	Las siguientes preguntas averiguan sobre tarifas a pacientes por servicios de atención de emergencia: Si un niño o un adulto sufre una lesión o enfermedad que requiera derivarlo a otro establecimiento con un nivel de atención superior, ¿el paciente o la familia tiene que pagar por el transporte o por el combustible?	Sí .... 1<br>No 0
3.2	Si un paciente de emergencia es derivado a otro establecimiento con un nivel de atención superior, ¿él/ella tiene que pagar la totalidad de sus cuentas antes de que él/ella pueda ser derivado efectivamente?	Sí .... 1<br>No 0

PESTAÑA 7: OFICIAL DE TRANSPORTE

Nombre Entrevistador ______________________________ Fecha (dd/mm/aa): ___ / ___ / ___

INSTRUCCIONES: Estas preguntas deben ser dirigidas al oficial de transporte del hospital.

1. DISPONIBILIDAD DE TRANSPORTE/MANTENIMIENTO/EQUIPO

INSTRUCCIÓN: Coteje las respuestas inspeccionando los vehículos disponibles.

1.1	Las siguientes preguntas averiguan sobre el vehículo(s) de transporte y las prácticas de transporte para referir pacientes a atención de mayor nivel: Para transporte de emergencia, el establecimiento ofrece... (lea cada ítem)	Sí	No
	a. ¿Al menos un vehículo actualmente operativo y disponible?	1	0
	b. ¿Al menos dos vehículos actualmente operativos y disponibles?	1	0
1.2	¿Con qué frecuencia se ejecuta mantenimiento preventivo?	A diario .... 3<br>Semanalmente .... 2<br>Mensualmente....................... 1<br>Menos de una vez al mes 0	
1.3	En los últimos 3 meses, ¿el vehículo de emergencia ha sido incapaz de transportar a un paciente de emergencia por falta de combustible?	Sí .... 0<br>No 1	

PESTAÑA 8: SUPERINTENDENTE MÉDICO

Nombre Entrevistador ____________________________ Fecha (dd/mm/aa): ___ / ___ / ___

INSTRUCCIONES: Estas preguntas deben ser dirigidas al superintendente médico del hospital.

1. SISTEMA DE TRIAJE

INSTRUCCIONES: Coteje las siguientes respuestas inspeccionando el protocolo de triaje regularmente usado.

1.1	Las siguientes preguntas averiguan sobre el sistema de triaje en este hospital: ¿El hospital tiene un protocolo de triaje para distinguir entre pacientes de urgencia y aquellos que pueden esperar?	Sí .... 1<br>No 0	
1.2	Este protocolo está en uso en las siguientes áreas… (lea cada ítem)	Sí	No
	a. Emergencia	1	0
	b. Otras áreas del hospital	1	0

2. INCIDENTES CON GRAN NÚMERO DE VÍCTIMAS

INSTRUCCIONES: Coteje las siguientes respuestas inspeccionando el protocolo de triaje para gran número de víctimas regularmente usado.

2.1	Las siguientes preguntas averiguan sobre la planificación en este hospital para gran número de víctimas: ¿El hospital tiene un plan vigente para gran número de víctimas?	Sí .... 1<br>No 0
2.2	¿Hace cuánto fue la última práctica que hizo el personal del hospital del plan de atención para un gran número de víctimas?	Dentro del último mes .... 1<br>Hace más de un mes 0
2.3	¿Las enfermeras Y los prescriptores están capacitados en el uso del plan para atención de un gran número de víctimas?	Sí .... 1<br>No 0

PESTAÑA 9: LISTA DE CONTROL DE LA ENCUESTA

Nombre Entrevistador ______________________________ Fecha (dd/mm/aa): ___ / ___ / ___

INSTRUCCIONES: Este inventario se debe ejecutar en la sala de emergencia luego de informar al personal senior de turno sobre su presencia. TODOS LOS MEDICAMENTOS/EQUIPOS/INSUMOS REGISTRADOS AQUÍ DEBEN SER VISTOS Y TODOS LOS EQUIPOS DEBEN SER PROBADOS O ESTAR EN USO.
Si usted no logra encontrar medicamentos o insumos, averigüe con el personal senior en servicio.

1. MEDICAMENTOS DE EMERGENCIA ESENCIALES

INSTRUCCIONES: Esta sección se refiere a las existencias de medicamentos en la sala de emergencia, no en la farmacia ni en el dispensario.

		Sí	No
1.1	¿Tiene la sala de emergencia los siguientes medicamentos ACTUALMENTE DISPONIBLES en stock?	Sí	No
	a. hidrocortisona IV	1	0
	b. salbutamol para inhalación (para nebulización)	1	0
	c. aminofilina IV	1	0
	d. oxígeno (cilindros o concentradores)	1	0
	e. adrenalina/epinefrina IV (1:1000)	1	0
	f. furosemida IV	1	0
	g. fluidos isotónicos: solución salina normal o Lactato de Ringer/Hartmann's	1	0
	h. fluidos de mantenimiento: dextrosa 5% en solución salina normal	1	0
	i. dextrosa (D10% o D50%)	1	0
	j. benzodiazepinas IV (PUEDE SER CUALQUIERA DE LAS SIGUIENTES: diazepam, lorazepam)	1	0
	k. barbituratos IV (PUEDE SER CUALQUIERA DE LOS SIGUIENTES: fenobarbital)	1	0
	l. sales para rehidratación oral	1	0
	m. antihipertensivos IV o sublinguales (PUEDE SER CUALQUIERA DE LOS SIGUIENTES: nitroglicerina, hidralazina, labetalol)	1	0
	n. aspirina PO	1	0
	o. atenolol PO	1	0
	p. dinitrato isosorbida PO	1	0
	q. heparina IV (o bajo peso molecular)	1	0

r. antimaláricos IV (cualquiera de: quinina, artemetro, o artesunato)	1	0
s. manitol IV	1	0
t. antimaláricos PO (artemetro/amodiaquina o artemetro/lumefantrina	1	0
u. ceftriaxona IV	1	0
v. co-amoxiclav IV	1	0
w. gentamicina IV	1	0
x. clindamicina IV	1	0
y. ciprofloxacina IV	1	0
z. metronidazol IV	1	0
aa. bencilpenicilina IV	1	0
bb. cloranfenicol IV	1	0
cc. aciclovir IV	1	0
dd. vitamina A PO	1	0
ee. antídoto veneno serpiente IV	1	0
ff. toxoide tetánico IM	1	0
gg. petidina IV	1	0
hh. tramadol IV	1	0
ii. diclofenaco PO	1	0
jj. paracetamol PO	1	0
kk. inyección lidocaína	1	0
ll. clorpromazina IM	1	0
mm. carbón activado	1	0
nn. ungüento ocular antibiótico (cualquiera de: eritromicina, tetraciclina)	1	0
oo. sulfadiazina de plata	1	0
pp. inyección de atropina	1	0
qq. Acetilcisteína	1	0
rr. vitamina K/fitomenadiona	1	0
ss. gel glucosa oral	1	0
tt. ampicilina IV	1	0
uu. amoxicilina PO	1	0
vv. cotrimoxazol PO	1	0
ww. eritromicina PO	1	0
yy. esteroides (PUEDE SER CUALQUIERA DE LOS SIGUIENTES: PO prednisolona, IV dexametasona)	1	0
zz. adrenalina/epinefrina IV (1:10 000)	1	0
aaa. prometazina	1	0
bbb. insulina regular	1	0

2. INSUMOS DE EMERGENCIA ESENCIALES

INSTRUCCIONES: Refiérase a la guía de fotos de equipos si algún equipo no le es familiar.

		Sí	No
2.1	¿La sala de emergencia tiene los siguientes consumes actualmente? disponibles Y funcionales?	Sí	No
	a. máscaras de oxígeno/ nebulizador – pediátrica	1	0
	a1. Máscaras de oxígeno/ nebulizador – adulto	1	0
	b. tubos de oxígeno	1	0
	c. tubos nebulizador	1	0
	d. vías aéreas oral/cánula Mayo o vías nasofaríngeas - pediátricas	1	0
	d1. vías aéreas oral/cánula Mayo o vías nasofaríngeas - adulto	1	0
	e. máscara de bolsa con válvula (ambú) -pediátrica	1	0
	e1. máscara de bolsa con válvula (ambú) -adulto	1	0
	f. cilindros de oxígeno	1	0
	g. laringoscopio	1	0
	h. hojas de laringoscopio – pediátrico	1	0
	h1. hojas laringoscopio – adulto	1	0
	i. tubos ET – pediátrico	1	0
	i1. tubos ET – adulto	1	0
	j. sondas pleurales/intercostal	1	0
	k. sondas de succión	1	0
	l. Yankauer/otra cánula de succión Rígida	1	0
	m. equipos/sondas de IV	1	0
	n. IV de gran diámetro (medida 18 o 16)	1	0
	o. IV tamaño pediátrico (medida 24 o 22)	1	0
	p. jeringas	1	0
	q. agujas – agujas adulto	1	0
	q1. agujas – agujas pediátricas	1	0
	q2. agujas – agujas para insulina	1	0
	q3. agujas – agujas intraóseas	1	0

r. Esfigmomanómetro – adulto	1	0
r1. Esfigmomanómetro – pediátrico	1	0
s. gasa	1	0
t. vendajes	1	0
u. cinta	1	0
v. férulas o yeso de París	1	0
w. agujas + sutura	1	0
x. fórceps/pinzas	1	0
y. porta aguja	1	0
z. sondas NG – adulto	1	0
z1. sondas NG – pediátrico	1	0
aa. collar cervical – adulto	1	0
aa1. collar cervical – pediátrico	1	0
bb. termómetro	1	0
cc. estetoscopio	1	0
dd. otoscopio	1	0
ee. oftalmoscopio	1	0
ff. bajalengua	1	0
gg. espéculo	1	0
hh. luz para examinación	1	0
ii. catéter/sonda de Foley – adulto	1	0
ii1. catéter/sonda de Foley – niño	1	0
jj. bolsa de catéter de orina	1	0
kk. mascarillas	1	0
ll. Guantes – desechables, no estériles	1	0
ll1. Guantes – estériles	1	0
mm. Glucómetro y tiras de prueba	1	0
nn. prueba de embarazo por orina	1	0
oo. prueba de orina con tira reactiva	1	0
pp. prueba rápido de Hemoglobina	1	0
qq. Sábanas para entablillar	1	0
rr. Cinturón pélvico	1	0
ss. Dispositivo de Succión/aspiradora (manual o máquina)	1	0
tt. agujas LP	1	0
uu. tubos Nasogástricos (NG) 24-42Fr	1	0
vv. balanza	1	0
ww. cronómetro o reloj mural con segundero visible en áreas de cuidado de pacientes	1	0
xx. oxímetro (separado de Dynamap)	1	0
yy. derivaciones de EKG	1	0

3. EQUIPO DE EMERGENCIA ESENCIAL (NO-consumibles)

INSTRUCCIONES: Esta sección se refiere a equipo estático (= no consumible) en la sala de emergencia.

		Sí	No
3.1	¿Tiene la sala de emergencia los siguientes equipos actualmente disponibles Y funcionales?	Sí	No
	a. dispositivo de succión/aspiradora (dispositivo manual o máquina en sala de emergencia)	1	0
	b. máquina nebulizadora (en sala de emergencia)	1	0
	c. concentrador de oxígeno (en sala de emergencia)	1	0
	d. EKG – un monitor de 12 derivaciones o 3 derivaciones (en sala de emergencia)	1	0
	e. máquina de ultrasonido (en sala de emergencia)	1	0
	f. máquina de rayos X (en hospital)	1	0

4. DISPONIBILIDAD DE TRANSPORTE/MANTENIMIENTO/EQUIPO

INSTRUCCIONES: El vehículo primario es el primero que el personal de llama de emergencia llama. El vehículo de transporte de emergencia primario debe ser inspeccionado e inventariado directamente para esta sección.

		Sí	No
4.1	¿El vehículo de transporte de emergencia primario tiene el siguiente equipo?	Sí	No
	a. ¿guantes?	1	0
	b. ¿sondas IV?	1	0
	c. ¿fluidos IV?	1	0
	d. ¿estetoscopio?	1	0
	e. ¿camilla?	1	0
	f. ¿camilla con inmovilizador espinal?	1	0
	g. ¿collar cervical?	1	0
	h. ¿vendas de presión?	1	0
	i. ¿férulas?	1	0
	j. ¿sacos de arena?	1	0
	k. ¿oxígeno?	1	0
	l. ¿tubos y máscaras de oxígeno?	1	0
	n. ¿inhalador de salbutamol?	1	0
	o. ¿esfigmomanómetro?	1	0
	p. ¿diazepam IV?	1	0
	q. ¿aspirina PO?	1	0
	r. ¿gel o dextrosa IV?	1	0

		Sí	No
	s. ¿glucómetro y tiras de prueba?	1	0
	t. medicamentos para el dolor (PUEDE SER CUALQUIERA DE LOS SIGUIENTES: diclofenaco, paracetamol, tramadol, petidina)?	1	0
	u. ¿laringoscopio y hojas?	1	0
	v. ¿tubos ET?	1	0
	w. ¿máscara de bolsa con válvula?	1	0
	x. ¿monitor cardiopulmonar?	1	0
	y. ¿máquina de EKG?	1	0

5. TRIAJE

5.1	¿Hay una estación de triaje en la clínica externa o en la sala de emergencia?	Sí ....0<br>No 1	
5.2	¿La sala de triaje está equipada con el siguiente equipo?	Sí	No
	a. ¿esfigmomanómetro?	1	0
	b. ¿estetoscopio?	1	0
	c. ¿termómetro?	1	0

6. REGISTRO DE CALIDAD (LIBRO DE REGISTRO DE SALA DE EMERGENCIA)

INSTRUCCIONES: Esta sección se refiere al libro de registro de la sala de emergencia o censo de pacientes, no a los registros médicos de pacientes.

6.1	¿El libro de registro de sala de emergencias tiene completas todas las filas y columnas?	Sí ....0<br>No 1
6.2	¿El libro de registro de sala de emergencias está actualizado?	Sí ....0<br>No 1

7. CALIDAD DE ENTREGAS DE PACIENTES

7.1	En términos de un sistema de entrega de pacientes, ¿están presentes los siguientes sistemas?	Sí	No
	a. Libro de rondas	1	0
	b. Pizarra blanca	1	0
	c. Planilla de egreso de pacientes	1	0
	d. Otro	1	0
